# A Rare Neuroendocrine Tumor at the Junction of the Cystic and Common Hepatic Ducts: The Value of a Multimodal Approach in Preoperative Diagnosis

**DOI:** 10.7759/cureus.74486

**Published:** 2024-11-26

**Authors:** Yoshito Saito, Masanori Ishida, Aoi Sukeda, Shuntaro Mukai, Yukari Wakabayashi, Elly Arizono, Yoshitaka Utsumi, Yuichi Nagakawa, Takao Itoi, Toshitaka Nagao, Kazuhiro Saito

**Affiliations:** 1 Radiology, Tokyo Medical University Hospital, Tokyo, JPN; 2 Pathology, Tokyo Medical University Hospital, Tokyo, JPN; 3 Gastroenterology and Hepatology, Tokyo Medical University Hospital, Tokyo, JPN; 4 Gastrointestinal and Pediatric Surgery, Tokyo Medical University Hospital, Tokyo, JPN

**Keywords:** common hepatic duct, computed tomography, cystic duct, endoscopic retrograde cholangiopancreatography, endoscopic ultrasonography, intraductal ultrasound, magnetic resonance imaging, neuroendocrine tumor

## Abstract

Neuroendocrine tumors (NETs) of the biliary tract are extremely rare due to a paucity of Kulchitsky cells. While their preoperative diagnosis remains challenging due to the lack of specific diagnostic markers and imaging findings, there have been no detailed reports describing the diagnostic utility of various imaging modalities for bile duct NETs at the junction of the cystic and common hepatic ducts. We report a case of a woman in her 40s who presented with jaundice and elevated hepatobiliary enzymes. Imaging studies identified a 17 mm × 15 mm × 13 mm nodule at the junction of the cystic and common hepatic ducts. Contrast-enhanced computed tomography (CT) demonstrated a well-enhanced nodule from the early phase, while magnetic resonance imaging showed slightly high intensity on T2-weighted images and strong diffusion restriction. Fluorine-18-fluorodeoxyglucose (18F-FDG) positron emission tomography/CT revealed mild 18F-FDG uptake with a maximum standardized uptake value of 3.5. Endoscopic retrograde cholangiopancreatography (ERCP) suggested extramural compression by a submucosal tumor, and endoscopic ultrasonography (EUS) and intraductal ultrasound (IDUS) revealed a well-circumscribed, homogeneous, hypoechoic submucosal nodule. This comprehensive multimodal approach allowed for the inclusion of NET in the preoperative differential diagnosis. The patient underwent cholecystectomy and extrahepatic bile duct resection, and histopathological examination confirmed a World Health Organization grade 1 NET at the junction of the cystic and common hepatic ducts. The patient remains recurrence-free after two years of follow-up. This is the first detailed report demonstrating the potential utility of a multimodal approach, including ERCP, EUS, and IDUS, in the preoperative diagnosis of biliary tract NET at this specific anatomical location, particularly in identifying the characteristic features of a submucosal tumor.

## Introduction

Neuroendocrine tumors (NETs) are tumors that originate from neuroendocrine cells widely distributed throughout the body. NETs can develop in various organs including the gastrointestinal tract, pancreas, and lungs [1]. According to the 2019 World Health Organization (WHO) classification (5th edition), NETs are classified into three grades - G1, G2, and G3 - based on mitotic count and Ki-67 index [2]. Higher grades indicate greater malignancy, and some NETs are associated with von Hippel-Lindau disease or multiple endocrine neoplasia type 1.

NETs are histologically well-differentiated tumors thought to originate from enterochromaffin cells in Lieberkühn's crypts or Kulchitsky cells [3]. Consequently, NETs rarely occur in the biliary tract where Kulchitsky cells are very scarce [4]. In addition to their low incidence, biliary NETs are difficult to diagnose preoperatively due to the lack of specific diagnostic markers and imaging findings and technical difficulties in obtaining adequate biopsy samples, due to the submucosal location. Although fewer than 200 cases of common bile duct NETs have been reported [5], there have been no detailed reports describing the diagnostic utility of various imaging modalities for bile duct NETs.

Here, we report a case of a middle-aged woman who presented with jaundice, where NET was included in the differential diagnosis of a distal bile duct tumor using a multimodal approach including conventional computed tomography (CT), magnetic resonance imaging (MRI), endoscopic retrograde cholangiopancreatography (ERCP), endoscopic ultrasonography (EUS), and intraductal ultrasound (IDUS), which was subsequently confirmed by surgery.

## Case presentation

A woman in her 40s was found to have jaundice during a regular visit to her primary care physician for hypertension treatment. Blood tests showed elevated hepatobiliary enzymes, and CT and MRI examinations revealed a tumor of the extrahepatic bile duct suspected to be causing obstructive jaundice. She was then referred to our tertiary care hospital (Tokyo Medical University Hospital) for additional diagnostic evaluation and treatment. Her medical history included only hypertension, and she was taking only antihypertensive medication. Family history revealed bladder and prostate cancer in her father and uterine malignancy in her mother. Physical examination showed jaundice of the skin and conjunctiva. Vital signs were stable. Initial blood tests at our hospital showed elevated hepatobiliary enzymes: aspartate aminotransferase 196 IU/L, alanine aminotransferase 408 IU/L, alkaline phosphatase 422 IU/L, gamma-glutamyl transpeptidase 683 IU/L. Total and direct bilirubin were elevated at 8.90 and 6.96 mg/dL, respectively. Tumor marker carbohydrate antigen 19-9 was slightly elevated at 58.9 U/mL, while carcinoembryonic antigen was within normal range. Hemoglobin, white blood cell count, and renal function were within normal ranges. Initial blood test results are shown in Table 1.

**Table 1:** Blood test results at the first visit to our hospital. Hepatobiliary enzymes and carbohydrate antigen 19-9 were elevated.

Parameters	Value	Normal range
White blood cell	3,800/μL	3,300-8,600/μL
Neutrophil	57.9%	42%-74.0%
Lymphocytes	32.0%	19%-47.0%
Monocytes	6.9%	2%-8.0%
Eosinophil	2.1%	<6.0%
Basophil	1.1%	<2.0%
Red blood cell	368 x 10,000/μL	386-492 x 10,000/μL
Hemoglobin	11.7 g/dL	11.6-14.8 g/dL
Platelets	236 x 1,000/μL	140-340 x 1,000/μL
Total protein	6.8 g/dL	6.6-8.2 g/dL
Albumin	3.5 g/dL	3.9-4.9 g/dL
Total bilirubin	8.90 mg/dL	0.2-1.2 mg/dL
Direct bilirubin	6.96 mg/dL	<2.0 mg/dL
Aspartate aminotransferase	196 U/L	8-38 U/L
Alanine aminotransferase	408 U/L	4-44 U/L
Lactate dehydrogenase	286 U/L	124-222 U/L
Alkaline phosphatase	422 U/L	38-113 U/L
Gamma-glutamyl transpeptidase	683 U/L	16-73 U/L
Blood urea nitrogen	6.8 mg/dL	8.0-22.6 mg/dL
Creatinine	0.45 mg/dL	0.40-0.80 mg/dL
Sodium	141 mEq/L	138-148 mEq/L
Potassium	3.3 mEq/L	3.6-5.2 mEq/L
Chlorine	106 mEq/L	98-108 mEq/L
Calcium	8.7 mEq/L	8.2-10.2 mEq/L
C-reactive protein	0.13 mg/dL	<0.30 mg/dL
IgG4 (latex agglutination)	22.2 mg/dL	11-121 mg/dL
Carcinoembryonic antigen	<2.0 ng/mL	<5.0 ng/mL
Carbohydrate antigen 19-9	58.9 U/mL	122-496 U/mL

**Figure 1:**
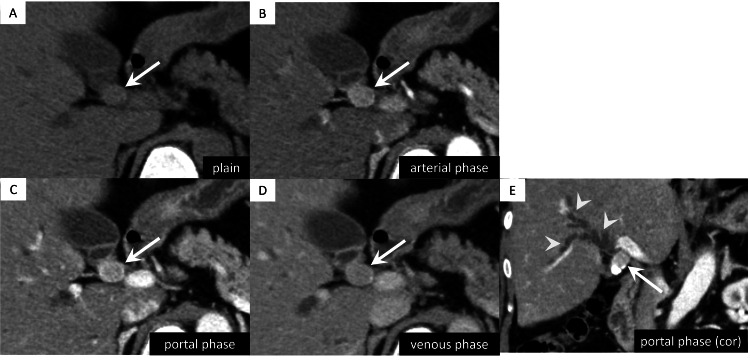
Abdominal computed tomography (CT) imaging findings. (A) Non-contrast CT showing a 17 mm × 15 mm × 13 mm nodule (arrow) at the confluence of the three bile ducts. (B-E) Dynamic contrast-enhanced CT demonstrating enhancement (arrows), peaking during the portal venous phase. The upstream of bile ducts are dilated (arrowheads).

Contrast-enhanced CT of the abdomen and pelvis revealed a 17 mm × 15 mm × 13 mm nodule at the junction of the cystic and common hepatic ducts that showed strong enhancement from the early phase (Figure 1).

**Figure 2:**
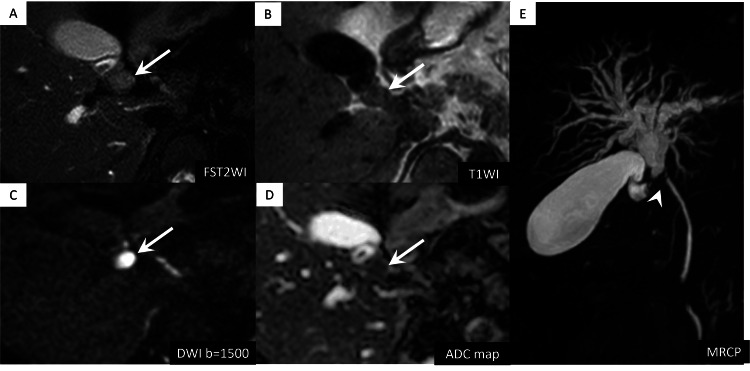
Abdominal magnetic resonance imaging (MRI) findings. (A) and (B) The nodule (arrows) shows mildly high signal intensity on T2-weighted imaging (T2WI) and low signal intensity on T1-weighted imaging (T1WI). (C) and (D) The nodule (arrows) exhibits marked diffusion restriction on diffusion-weighted imaging (DWI) and the apparent diffusion coefficient (ADC) map (0.63 × 10⁻3 mm²/s). (E) Magnetic resonance cholangiopancreatography (MRCP) shows dilation of the common hepatic duct and intrahepatic bile ducts upstream of the nodule. Bile duct compression by the nodule produces a signal defect (arrowhead).

The upstream of common hepatic duct and intrahepatic bile ducts were dilated. The gallbladder was neither enlarged nor distended. On unenhanced abdominal MRI, the nodule showed slightly high intensity on T2-weighted images, low signal intensity on T1-weighted images and strong diffusion restriction (Figure 2).

**Figure 3:**
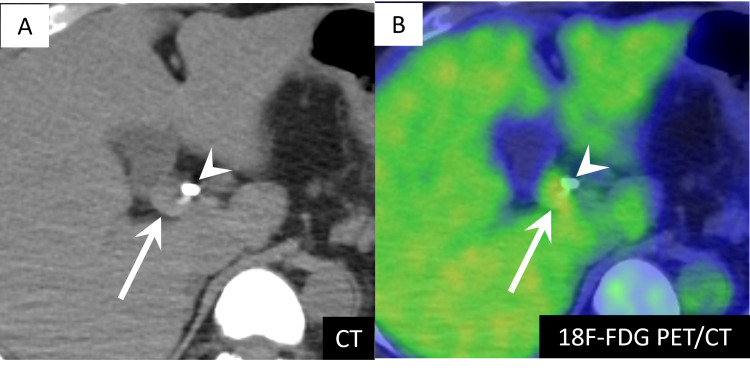
Fluorine-18-fluorodeoxyglucose positron emission tomography (18F-FDG PET)/CT imaging findings. (A) Non-contrast CT shows a nodule (arrow) at the confluence of the three bile ducts. A bile duct stent is inserted in the common bile duct (arrowhead). (B) Mild FDG uptake with an SUVmax of 3.5 is observed in the nodule (arrow). The common bile duct stent is also observed (arrowhead).

Magnetic resonance cholangiopancreatography showed dilation of the common hepatic and intrahepatic bile ducts, similar to CT findings. Fluorine-18-fluorodeoxyglucose (18F-FDG) positron emission tomography/computed tomography showed mild uptake in the nodule with a maximum standardized uptake value of 3.5 (Figure 3).

**Figure 4:**
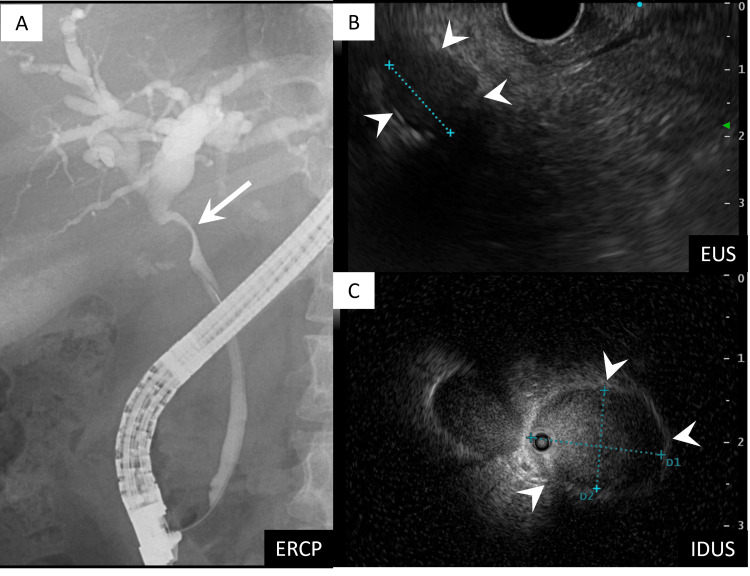
Findings of endoscopic retrograde cholangiopancreatography (ERCP), endoscopic ultrasound(EUS), and intra-ductal ultrasound (IDUS). (A) ERCP shows a stenotic lesion at the confluence of the three bile ducts, caused by extrinsic compression resembling a submucosal tumor (arrow). (B) and (C) EUS and IDUS reveal a well-demarcated, smoothly margined nodule with heterogeneous hypoechoic features (arrowheads).

Suspecting a bile duct tumor, ERCP was performed (Figure 4). It showed bile duct stenosis at the junction of the cystic and common hepatic ducts, suggesting extramural compression by a submucosal tumor. EUS and IDUS revealed a well-circumscribed intramural nodule with smooth margins and heterogeneous low echogenicity, suggestive of a submucosal tumor (Figure 4).

ERCP, EUS, and IDUS findings suggested that the nodule did not originate from the bile duct epithelium. In other words, a submucosal tumor was considered more likely than cholangiocarcinoma. The differential diagnosis for bile duct submucosal tumors included NET, schwannoma, leiomyoma, and gastrointestinal stromal tumor. Forceps biopsy of the stenotic region and bile cytology during ERCP were performed, but histopathological examination showed no evidence of tumor lesions.

**Figure 5:**
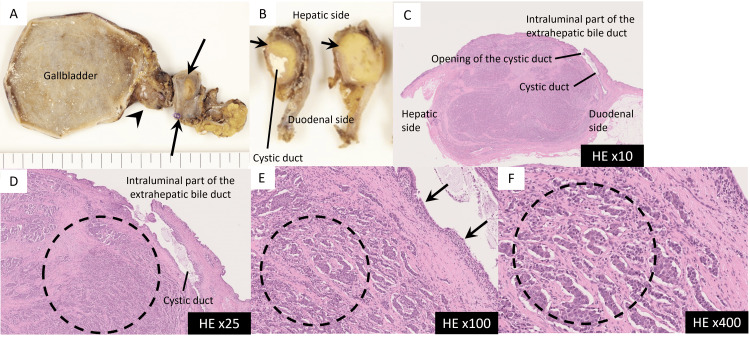
Hematoxylin and eosin (HE)-stained histopathological specimens. (A) and (B) A well-demarcated, 2 cm yellowish-white nodule (arrows) is observed in the extrahepatic bile duct, just below the cystic duct (arrowhead) orifice. (C)-(F) Tumor cells with mildly pleomorphic, enlarged round nuclei and pale eosinophilic cytoplasm are arranged in trabecular patterns or small clusters (dotted circles).

Subsequently, cholecystectomy and extrahepatic bile duct resection were performed. Macroscopically, the tumor appeared as a well-circumscribed, approximately 2 cm, yellow-white nodule in the common hepatic duct just upstream of the cystic duct junction, with part of it extending into the cystic duct (Figure 5). Microscopically, using hematoxylin and eosin staining, tumor cells with slightly irregular round enlarged nuclei and pale eosinophilic cytoplasm formed cord-like arrangements and small clusters (Figure 5).

**Figure 6:**
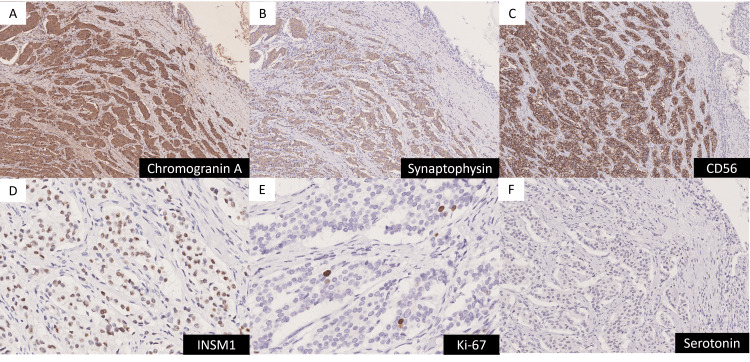
Immunohistochemical staining. (A)-(D) Immunostaining was positive for chromogranin A, synaptophysin, CD56, and INSM1 (stained brown). (E) The Ki-67 labeling index was less than 1%. (F) Immunostaining was negative for serotonin.

The tumor primarily proliferated in the hilar bile duct region on the hepatic side of the cystic duct junction and had invaded the cystic duct wall. Tumor invasion extended to the subserosal layer, with positive surgical margins. Immunohistochemically, tumor cells showed positive staining for synaptophysin, chromogranin A, CD56, and INSM-1, while being negative for serotonin. The tumor demonstrated very low proliferative activity, with a Ki-67 labeling index of less than 1% and a mitotic figure count of less than 1 per 2 mm² (Figure 6).

Serotonin staining was negative. No lymph node metastasis was found in the dissected hepatoduodenal ligament or common hepatic artery trunk territory. Based on these findings, the tumor was diagnosed as a WHO grade 1 NET at the confluence of the junction of the cystic and common hepatic ducts. The postoperative course was uneventful, and the patient was discharged without complications. There has been no recurrence during two years of follow-up.

## Discussion

Biliary tract NETs are extremely rare. In Modlin et al.'s analysis of 13,715 gastrointestinal NET cases, the incidence of biliary NETs was only 0.32% [6]. Compared to cholangiocarcinoma, biliary NETs occur more frequently in women and younger patients [7]. Regarding location, they most commonly occur in the common hepatic and distal bile ducts (19.2%), followed by the middle bile duct (17.9%), cystic duct (16.7%), and proximal bile duct (11.5%) [8]. Other tumors that can occur in the extrahepatic bile ducts besides NET include cholangiocarcinoma, cholangiocellular adenoma, leiomyoma, paraganglioma, lipoma, and granular cell tumor [9]. Differentiating these tumors from cholangiocarcinoma, which is particularly common, is important because it affects procedures such as right-extended hepatectomy.

Generally, NETs in any part of the body characteristically appear as well-defined hypervascular masses on contrast-enhanced CT. On MRI, they typically show lower signal intensity than liver parenchyma on T1-weighted images, higher on T2-weighted images, and restricted diffusion [10]. While the MRI findings of our case were consistent with these characteristics, cholangiocarcinoma can show similar CT and MRI findings, making it difficult to differentiate between the two based on CT/MRI alone. As a reference, typical CT and MRI findings of cholangiocarcinoma include irregular ductal wall thickening, delayed enhancement, and upstream biliary dilatation. The relatively mild 18F-FDG uptake suggested a low-grade malignancy if malignant, but this finding was not sufficient to rule out cholangiocarcinoma. Additionally, the treatment approaches for cholangiocarcinoma and NET differ significantly - while surgical resection followed by adjuvant chemotherapy is the standard treatment for cholangiocarcinoma, NETs require a multimodal approach including surgical resection when feasible, somatostatin analogs, targeted therapies, and/or peptide receptor radionuclide therapy depending on tumor grade and stage.

In this case, ERCP, EUS, and IDUS were able to capture findings of a submucosal tumor different from typical cholangiocarcinoma. In particular, the EUS and IDUS findings showed characteristics of a submucosal tumor, lacking features typical of biliary epithelial tumors such as destruction of normal bile duct wall echo layers, eccentric wall thickening, and invasion of adjacent tissues or vessels [11]. The ability to demonstrate findings suggestive of non-epithelial tumors through ERCP, EUS, and IDUS examinations helped include biliary NET, which develops submucosally, in the differential diagnosis. Typically, non-epithelial tumors of the bile ducts present with the following findings. ERCP revealed smooth extrinsic compression of the bile duct with a preserved mucosal pattern. EUS showed a well-defined, homogeneous, hypoechoic mass within the bile duct wall. IDUS demonstrated a homogeneous hypoechoic tumor continuous with the second (submucosal) layer while the first (mucosal) and third (fibromuscular) layers remained intact, consistent with a submucosal tumor.

Biopsy of bile duct tumors can also be performed during ERCP and EUS. EUS-guided fine-needle aspiration has been reported to be potentially useful for preoperative histological diagnosis of malignant tumors [4], with case reports of successful diagnosis of well-differentiated biliary NET by EUS-guided fine-needle aspiration [12]. However, such successful diagnostic cases using these methods are limited, and they cannot be considered standard methods for preoperative diagnosis. Since NETs are submucosal, the tumor often does not extend to the mucosal surface, resulting in a high false-negative rate [4,13]. In this case, as well, ERCP cytology and EUS tissue biopsy did not lead to diagnosis, consistent with previous reports. It should be noted that tissue sampling may not directly lead to diagnosis even when biliary NET is suspected.

Although rare, NETs can potentially secrete hormones such as serotonin, insulin, gastrin, somatostatin, and glucagon [14]. Histologically, fibrosis in NETs is reportedly associated with serotonin immunoreactivity in the pancreas [15]. In this case, although submucosal fibrosis was observed, serotonin staining was negative. No reports of serotonin-positive biliary NET could be found in PubMed.

The first-line treatment for biliary NET is surgical resection when resectable. Since approximately 20% of patients with biliary NETs have lymph node metastases [13], lymph node dissection is necessary. Although this case had no lymph node metastases, careful follow-up is necessary for the possibility of local recurrence due to positive surgical margins. While not conforming to current NET classification, previous reports demonstrate relatively favorable outcomes following R0 resection (complete tumor resection with histologically negative margins), with five-year and 10-year survival rates of 84% and 80%, respectively [16].

## Conclusions

Biliary NET is an extremely rare disease and difficult to diagnose preoperatively due to the lack of specific diagnostic markers. In this case, using ERCP, EUS, and IDUS enabled us to include NET as a differential diagnosis of a bile duct submucosal tumor.
